# Pathological Description of Continued, Remittent, and Intermittent Drunkenness

**Published:** 1862-07

**Authors:** O. M. A. Salvatori


### Art. VII. — PATHOLOGICAL DESCRIPTION OF CONTINUED, REMITTENT, AND INTERMITTENT DRUNKENNESS.

By O. M. A. Salvatori.

READ BEFORE THE IMPERIAL PHYSICO-MEDICAL SOCIETY OF MOSCOW, 8TH DECEMBER, 1817, AND TRANSLATED FROM THE SECOND VOLUME OF THE SOCIETY'S TRANSACTIONS, BY W. B. TURNBULL, ESQ.; F.S.A., SCOT.; HON. MEMB. ROY. SOC. ANTIQ. OF COPENHAGEN; CORRESPONDANT DU COMITÉ IMPÉRIAL DES TRAVAUX HISTORIQUES ET DES SOCIÉTÉS SAVANTS DE FRANCE, ETC. ETC.

[We gladly avail ourselves of an opportunity afforded by the kindness of Mr. Turnbull of laying before our readers a translation of Salvatori's celebrated dissertation on Drunkenness—a dissertation little known in this country from the great scarcity and difficulty of access of the volume in which it originally appeared.

This essay is of rare interest to the medical scholar, not only because in it habitual drunkenness was probably for the first time treated nosographically, but also on account of the singularly able and philosophical investigation of the subject, which Dr. Christison states is the ablest he has yet seen.*

With Dr. Christison, "we have some doubt of the stability of the author's very numerous radical cures," and we have also "even less faith in the virtues of his Serpyllum." "It is a pity, for the sake of the disciples of the temperance movement," writes Dr. Christison, "that Salvatori's repellent antidote should remain unknown. He calls it *Serpyllum,* and gives no account of it for

---

* *On some of the Medico-Legal Relations of the Habit of Intemperance.* Edin. 1861. Pp. 52.

its identification. I could not imagine such potency in the only serpyllum of my acquaintance, the pretty little purple-flowered wild thyme which abundantly clothes Arthur's Seat, and other trap hills around this city, and which belongs to a natural family of plants of singularly uniform, simple, well-ascertained properties. But such it is. For *Sir James Wyllie*, in the edition of his *Pharmacopœia Castrensis Ruthenica*, or Russian Army Pharmacopœia for 1840, admits into his list the *Thymus Serpyllum*, with the information that Linnæus recommends it for the headache which follows a debauch, and Salvatori as a cure for the insane craving for strong drink." Dr. Christison adds, that he does not feel bound to subscribe entirely to Salvatori's pathology, simple though it be as well as ingenious. "But," he writes, "all my experience, together with that of every esteemed member of my profession with whom I have conversed on the matter, coincides with his doctrine as to the fact [in which we fully concur]. That there is an *Anamethysis*, or drink-craving—a disease and not a mere aggravated vice—which may betray itself too suddenly to admit of its having arisen as a simple immorality—and of which mere vicious excess, when it is the cause, acts in general as a predisposing cause only:—that whatever opinion may be formed of its real essence, or pathological cause, its manifestation is a form of unsoundness of mind—an insane avidity for liquor—an insane "drink-crave"—drink-madness. To the proof supplied by Salvatori, I may add, that sometimes family constitution has much to do with it, as with other insanities. Either we see the craving itself prevail in particular families—a fact which the physicians of assurance companies know well, and which is a guide to all prudent assurance directors, or the family history presents a liability to affections of the head in general—to apoplexy, palsy, epilepsy, inflammation of the brain, and ordinary insanity—diseases which equally point to a family constitutional predisposition."*—Ed.]

The disease to which I desire to call your attention, hitherto banished from nosological practice, is one which if not entirely neglected by physicians, yet seems to have been considered as one impossible to be combated and overcome by ordinary remedies. Since, however, no disease more pernicious, or one more fatal can afflict humanity, I cannot doubt that the studies which I have directed towards the knowledge and cure of this disease will meet with impartial judges, who will accept what I venture to propound as an experiment rather than as a completed work.

I speak of the immoderate drinking of vinous and ardent fluids, and that peculiar disease arising therefrom, which may be

* *Op. cit.*, p. 53.

termed *furor bibendi;* or, to employ a Greek term, οἰνομανια. Instances of this singular insanity are to be met with everywhere; for who does not lament some friend, companion, domestic, or other useful citizen, a victim to it?

Although nosologists appear to have neglected this disease, yet pathologists in their description of maladies have remarked the evil consequences of excessive drinking, which apparently have been by no one more carefully and critically described than by the immortal Gaubius, in his *Institutes* (§ 467 and 468), who remarks, that "Wines, old malt liquors, chiefly the drier kinds, and ardent fluids distilled from them, whether simple or compounded, possess a spirituous acridity created by fermentation; for it acts by thickening and inspissating the fluids, by hardening the firm parts, stimulating the fibres, and by exciting the animal impetus, driving into confusion the vital and sentient powers. Wherefore it follows that immoderate drinking occasions thirst, intoxication, accelerated circulation, increased heat, rarefaction of the humours, dissipation of the more subtle properties, coagulation of the humours, inflammatory disposition of the blood, constriction of the testicles, dilatation, rupture, local errors, obstructions, inflammations, but also inordinate wearying and depressing commotions of the nervous system, with excessive determination of humours to the head, whence trouble of the encephalon and repletion, and afterwards, all stimulation having passed off and given place to collapse, subsidence of humours, shrinking of vessels, torpor of the circulation, coldness, exhausted powers, languor of every function, and a train of symptoms the very opposite to those which existed at first.

"By duly considering these effects, what daily happens to drunkards may be easily understood. Why do fresh potations remove the depression caused by the debauch of the previous evening? Why does this abuse from daily habit become a necessity, and seldom or never be disused, but rather gradually increases from the mildest to the strongest form? Why is it so much more injurious to the young than to those of advanced years? Why does it so weaken the digestive functions, that all appetite for food being lost, life is sustained solely by fluids? What should change the humours, constantly fed by such a quantity of spirits, into sluggishness? Whence, in drunkards, cachexy, consumption, leucophlegmatic habits, and dropsy? Whence redness of the eyes and pimples of the face? Why are they inclined to acute and inflammatory diseases, and by such so dangerously affected? Why are they subject so often to trembling, paralysis, apoplexy, and other derangements of the senses and motions?"

In this excellent exposition nothing seems to be wanting, except that the very necessity of drinking, wherein this abuse, as the author says, arises from daily habit, should be treated as a distinct disease. Had the author done so, perhaps, long since its prevention and cure might have been known.

In like manner as fevers are divided into ephemeral, continued, and intermittent, so also drunkenness, which Cicero says is derived from drink, as lover from love, is divided into ephemeral, continued, and intermittent; all being gradations of the same vice.

*Ephemeral* drunkenness is that which either daily, or if not daily, yet frequently occurs, but in such manner as he who is addicted to it does not get drunk continuously, but occupies himself during a certain portion of the day, devoting solely his leisure hours to Bacchus. To such a custom Anacreon is said to have been addicted, and many others whom

—— "the ivy, fit reward
To grace the brow of learned bard,
Equals with the gods above;
Whom the cool and quiet grove,
The nymphs and satyrs sylvan dance
From the vulgar crowd advance.

Which, indeed, I should not presume to call a disease that seems to be averted,

"If Euterpe nor refuse
To lend her pipe to aid the Muse,
Nor Polyhymnia disdain
Her Lesbian lyre to tune again."—*Robinson.*

But others are more unfortunate. From daily habit arises the necessity of drinking, a real and peculiar disease—*continued intoxication*, which the Greeks perhaps would have called *ἀναμέθυσις*. They continually drink by day and by night, nor suffer the drunkenness, by continued swilling, to be abated before the humours, by enormous quantity of spirits, being changed into a sluggish condition, the nervous system wholly relaxed, and the powers of digestion ruined, they reject whatever else they swallow. Thus corrective nature averts the destruction of the body, oppressed by intoxicating fluids, the acrid matter collected in the stomach is evacuated upwards and downwards; sleep returns, the prostrated and diminished powers of body and mind are by degrees restored, again, in a short time, to be oppressed by the same insanity.

Thus, therefore, by the resistance of nature herself, death being averted, are produced remissions and *intermissions* of continued drunkenness, sometimes longer, sometimes shorter, sometimes more complete, at others less so. For the stronger they are in mind and body, and the more rarely occasional causes provoking to intoxication occur, the longer they generally abstain from the repeated abuse of liquor.

What, therefore, I have called *continued* and *intermittent* drunkenness, differ from each other in degree rather than in character.

Truly continued drunkenness, in the stricter sense of the term, seems to be inadmissible by nature; for the body, unless the disturbing cause was occasionally interrupted, must necessarily in a short time perish. But more complete abatement effects intermission, which itself in confirmed drinkers, although they abstain from intoxicating liquor, is so imperfect that even their sobriety appears to be drunk.

That this continued, remittent, or intermittent drunkenness may, in all respects, be termed a *disease*, is proved by the fact that the parties are compelled to drink against their will. Nothing can restrain them: neither the prayers of friends, the tearful entreaties of wives or children, nor the imminent ruin of their private affairs. They bewail their miserable estate, and denounce the dire necessity that controls their will as a punishment inflicted by heaven. But whether this necessity is to be ascribed to a perverted intellect, or a preternatural condition of body, or whether, therefore, drunkenness is to be considered a mental or bodily disease, it is in all respects proper to inquire.

Although in drunkards we find the intellect generally weakened and injured, yet we do not find it so perverted that it must necessarily rush to destruction by itself. We not unfrequently see men who are accustomed to direct all their other actions prudently and rationally, afflicted with this plague-spot. Hence it can scarcely be doubted that it lies in the body, and that the proximate cause of the disease exists not in the perverted condition of the mind, but in the body, whereby the mind itself is weakened and by force of the same cause impelled to insanity.

Wherefore, as far as I can, I shall endeavour to contribute to a knowledge of this most fatal disease, by setting forth what I have been able to discover of its causes, symptoms, and effects.

The external or occasional causes of this disease are of a twofold character—namely, *psychical*, as primarily affecting the mind; and *physical*, which direct their first force in the body. Of the first class are melancholy, grief, uncertainty, inert sloth, despair; in a word, all things that induce constant languor and depression. To the other class I refer *tænia*, which I have found to be a frequent cause of drunkenness in Siberia, as it was formerly found in Moscow by the eminent physicians, Skiada and Minderer.

Since the bodies of men are so constituted, that the same external causes have different effects in different bodies, *predisposing causes* have to be defined, which affect such constitution, that the occasional cause being before us we may derive from it the disease.

The principal of these seem to me to be—1st. A strong incitable nervous system; for daily observation shows that men excelling in body and mind are more disposed to drunkenness than those of a feeble habit. Weaker persons are very much affected by strong liquor, therefore they abstain; and it is a fact that those who are affected with violent headache after drinking, as well as those again who are not easily intoxicated, seldom become drunkards. 2nd. Another predisposing cause is in the frequent abuse of wine, and that drunkenness which I have called ephemeral; for thereby that excessive incitability of the nerves seems to be created, which appears to be the chief seat of the disease. 3rd. Finally, what appears to generate this disease is a weak mind, disposed rather to imagination than reflection, an internal capacity oppressed by the use and abuse of reason, no religion or an erroneous one, referring piety rather to works of expiation than to a virtuous rule of life.

Observation readily suggests these occasional and predisposing causes; but the question is more difficult as to the *proximate cause* of the disease, whether such proceeds from internal change of the body, whence that fury and necessity of drinking in which we see the disease consists, is produced.

That in itself comprehends these two questions:—

1st. Why in the time of intermission does the drunkard return to the use of liquor?

2nd. Why should the drunkard continue to swill?

Since these can scarcely be answered without a thorough knowledge of every symptom, after describing the course and symptoms of the disease, I shall return to the resolution of the question of the proximate cause.

The principal symptoms of continued drunkenness are these: the fasting stomach in the morning is annoyed by nausea, languor, vertigo, frequent eructation, a more copious secretion of saliva, tremor of the limbs, and general uneasiness.

After a few such days are passed, along with the physical powers, appetite for food becomes prostrated; the sleep is short and unquiet; strange melancholy succeeds, so that nothing seems more intolerable than life. In this very sad state, the image of his accustomed potation presents itself to the mind of the wretched individual, and this alone so occupies and disturbs it, that it can in nowise receive or retain any other impression. Therefore all unavailing are the arguments and advice of friends, or the entreaties and tears of his family; the miserable man feels that he is compelled by a dire necessity to drink, and professes that if it is denied to him he will go mad. Impelled by this fearful necessity, he puts the cup to his lips, and by the first draught becomes intoxicated, if he uses spirits ; if he drinks wine, a larger quantity is required.

Presently the former troublesome symptoms vanish ; the melancholy is dispelled ; with the reviving liquor the nausea of the stomach passes off ; *coma vigil* ensues ; the powers of the body revive ; the pulse is rapid and subfebrile. For the first two or three days the mind is cheerful ; but afterwards it begins to be irritable, and becomes very irascible if liquor is not at hand. Objects frequently appear to be double, distant ones are indistinctly seen, the eyes are not closed but distracted, the speech becomes thick, the mind itself is alienated ; then melancholy succeeds, the victim bewails his wretched lot ; the pulse becomes spasmodic. The course of intoxication being then complete, he vomits a glutinous, acid, and almost corrosive matter ; he abhors the liquor ; the mind returns, self-accusing. To the vomiting succeed languor, prostration of strength, comatosity, profuse perspiration during sleep, with a spirituous odour and smell in those who use diluted alcohol as an intoxicator, pain in the stomach, diarrhœa with tenesmus, and an acrid and viscous matter similar to what was vomited, a weak and almost vermicular pulse, severe headache, thirst, tremor of the hands—more vehement when pure water is offered for allaying the thirst. This course of accesses and order of symptoms is observable in those who make use of wine or spirits as an intoxicator. But those who get drunk with *vini sicera* (dry wine) or beer, or *sicera dulci* (sweet wine), or bitters, vomit frequently and with great effort.

I have never seen the duration of such an access exceed fourteen days : sometimes it terminated on the sixth, at others on the ninth day. The sober intermission that follows, wherein both the usual intoxicator and anything spirituous is detested, varies in duration. I have observed, however, that those for the most part who were drunk for a fortnight, remained sober for another fortnight ; others abstained for a month, others for half a year.

It seems to be worthy of note, that those who are addicted to drink remember when it is over all that passed during the entire paroxysm ; but the thoroughly drunk are generally oblivious of the greater part that happened to them while in that state.

Many, perhaps, might believe that the *tænia*, which I have previously pointed out as an occasional cause of inebriety, would be killed by the quantity of spirits imbibed ; but, on the contrary, I have seen the same rather nourished and increased to an enormous length in drunkards. Drunkards who, at the end of the access, are more thoroughly cleared out above and below by perspiration and urine, enjoy a longer intermission.

At the commencement of the intermission there is a desire for acids, such as cranberries, pickled cucumbers or cabbage ; then, till the end of the intermission, health, appetite, and digestion, are good enough ; but at the end of the interval the digestion is affected, and the unfortunate individuals are again drawn into a vast gulf. In drunken or oinomaniacal females, the menstrual flux is inordinate and excessive, or amenorrhœa is observed. Sometimes I have seen the *furor bibendi* united to *furor uterinus* ; so that in place of melancholy, which usually precedes accesses of intoxication in men, nymphomania occurred. In such the accesses observed the catameniacal period.

The principal effects of chronic drunkenness are the following, as noted by Avicenna :—1st, disorder of the liver by resolution of its own heat; 2nd, weakness of the brain, and also, 3rd, of the nerves; 4th, spasms; 5th, paralysis; 6th, sudden death. I may add, the viscera united to the *vena portarum* are more affected than other parts, chiefly in those who abuse wine and spirits; hence obstructions in the belly, dropsy, and tetters arise. They who get drunk upon beer become stupid, and fall into paralysis, impotence, apoplexy, or madness. Drunken women, becoming pregnant, bring forth dead children, frequently with ulcerated skin. But if I wished to describe all the consequences of this disease, I should require to write an Iliad. Yet there are some drunkards who, except into drunkenness, almost never fall into any other disease.

With reference to *prognosis* it is in the first place to be observed, that drunkenness is an affection which the healthy bodies of drunkards sometimes resist for an incredible time. I have seen a man of eighty, who had been a drunkard from his thirtieth year;. and another constantly so from his eighteenth until his fortieth year, when I cured him of his *furor bibendi.*

But those who are interrupted in accesses of intoxication, lest they should give way to their depravity, by taking emetics or anything else that provokes nausea, frequently fall into serious illnesses and peril of their lives.

Mental complaints are occasionally cured by a strong affection, and the like sometimes is wrought by a great change of the body, such as typhus fever, for example, brings about.

The more copious the evacuations are at the end of accesses, the greater is the hope of a cure.

Women are generally less easily cured than men.

Having now given the history of the whole disease from practical observation, I shall revert to the important question previously mentioned, of its *proximate cause.* And, in the first place, let us attend to those symptoms which are noticeable in the beginning of the paroxysms: languor, melancholy, difficult digestion, want of appetite for food, and the insuperable desire for drink.

These symptoms appear to me to indicate a disease which has its seat in that part of the nervous system which lies under the hypochondrium, and which used to be called the system of abdominal ganglions. Therefore, continuous drunkenness ought to be classed as a species of that widely-spread genus of diseases, which are commonly called hypochondriacal and hysterical. Such the symptoms of the disease, its predisposing causes, and those occasional ones that nourish and make it constant, direct it ought to be. For it may be supposed that those intoxicating fluids which exhibit such a power on the common sensorium, can scarcely exercise less on the abdominal cerebrum. Therefore by repeated and continued excitement and perturbation, the abdominal nerves, being weakened, vitiated, injured, and rendered unfit to perform those functions which consist in regulating the work of digestion and secretion, must be disposed to an appetite for what is noxious.

The like is seen to happen to infants whose abdominal nerves are injured by unwholesome meat or drink. From a similar cause, with other increasing evils, arises that prodigious voracity and hankering, principally for injurious things, nourishing and increasing the present vice. The same we daily see happens to hypochondriacs and hysterical people.

To the same estimate of the disease, a periodical and regular *type* which is generally to be remarked in it, also conduces. For such is the law of the abdominal organs, that they usually observe in their motions a certain type, and a certain intermission in morbid affections.

I therefore think that the proximate cause of drunkenness lies in the preternatural exhausted state of the abdominal nerves, disturbed perhaps alike in fabric as in function.

But besides this, there seems to be another cause, which probably proceeds from the former, although it has less power in creating the disease, and which, I think, deserves not less attention. Namely from causes provoking and nourishing, predisposing and occasional, the humours also are depraved, the secretions of the abdominal viscera are impeded and perverted, so that not only do evil humours circulate through the entire body, but likewise—and chiefly in the stomach—deposits noxious, irritating, and inimical to the nerves are formed. Therefore, I think that to this, in addition to the depraved state of the nerves, chiefly abdominal, the whole disease is to be ascribed, and that against both of these causes every method of cure should be directed.

If these observations and ideas be deemed at all worthy of the attention of the Society, I shall in a future meeting mention those remedies which I have found most frequently productive of a complete cure, together with an account of each individual case.

### THERAPEUTIC DESCRIPTION OF CONTINUED, REMITTENT, AND INTERMITTENT DRUNKENNESS.

By O. M. A. SALVATORI.

(*Read before the same Society*, 12*th January*, 1816.)

As to-day I desire to submit to you a method for curing oinomania, I had proposed, in the first instance, to communicate all the observations which are the result of the daily practice of the last five years. But as the number of cases amounts nearly to fifty, I shall not trespass upon your patience with more than three; leaving to a future opportunity, if it shall be agreeable to the society, the remaining cases worthy of attention.

### CASE I.

M. N., a handsome woman, about thirty, of sanguine temperament, robust habit of body, fat, snowy skin, fair hair, acute and sharp-witted, delicately brought up in a noble family, was freed from scrofula which had afflicted her youth on the appearance of the catamenia. Married at twenty-one, to a feeble man, and become pregnant in the third month of her marriage, she had a very laborious period. After her delivery, she left her native city to accompany her husband, who had been appointed to a public office in a remote place. There, discontented with domestic seclusion, and looking back to her former life spent in society and gaiety, she became acquainted with some of her own sex who were addicted to their cups, and by degrees acquired a habit of drinking spirituous liquors. At first headache, the result of each excess, suggested caution and moderation, which, by bad advice, were rejected for a very different prescription :—

"If next morn your head feels the carouse over night,
Take another stiff glass and 'twill set you all right."

During this dissipated mode of living, she again became pregnant, but, by reason of her frequent excesses, aborted in the third month, after which her menstruation began to be irregular and leucorrhœa ensued. When the husband discovered that his wife was devoted to drinking, he at the same time found that she was transported with lust; which, as on account of his constitution he was unable to satisfy, and moreover experienced the fury and anger of his wife, whom he justly reproached, he sorrowfully thought himself obliged to indulge her in these excesses. Thus, when nothing restrained this dissolute woman out of this daily intoxication, at the end of some months continued intermittent oinomania proceeded, recurring monthly at the time of the catamenia. In this, after she had continued for ten successive years, both the duration of the accesses and the vehemence of the *furor uterinus* gradually increased, until at length, during the paroxysm, she threw aside all modesty and decency; on the expiry of which she was so penitent and ashamed of her obscene behaviour, which she remembered perfectly, that she would be dissolved in tears, and abstain from the benefit of the Church, which she believed would be profaned by her entering it, not knowing whence she might expect salvation. But on the access of a new paroxysm, penitence, modesty, and resolution to abstain, once more took flight.

The physicians who were accustomed to administer to her other complaints, attributing the *furor uterinus* and oinomania with which she was afflicted to mental, not to bodily defect, attempted no remedy, but left the unfortunate woman to her luckless fate. She first sought my advice for the copious *fluor albus* and menstrual menorrhagia which prevailed with her on the following day. I prescribed essence of assafœtida, *liquor c. c. succinatum,* and *elixir acidum Halleri* with infusion of milfoil. This was on the 24th of November, the day preceding her usual time of anamethysis. Again called in on the 27th of the same month, I found her in bed, complaining of congestion in the head, heartburn, rumbling in the intestines, pain of the lower region of the belly, thirst, melancholy, vigilance, torpor of the thighs. Menstruation had not yet appeared, and she felt internal uneasiness. I considered it advisable to substitute tincture of saffron with *liquor anodynus mineralis Halleri elixir.* On the following day, 28th November, a few symptoms showed themselves, and on the 29th the menses made their appearance with a severe colic. Whereupon, on the 30th, I ordered her to remain in bed, continue the same medicine, and drink camomile tea. On the 1st December, the menses flowed freely, continued on the 2nd, and she felt herself much better. On the 3rd she rose; the flow had almost entirely ceased, and she felt cheerful. On the 4th she was extremely well; the *fluor albus* following the menstruation, which had been more than customary, was moderate; however the medicines were continued till the 7th, when she thanked me with very grateful and complimentary expressions, believing herself to be cured of the inebriety which for that time had ceased. I prescribed *tinctura ferri acetici œtherea* with infusion of milfoil, which she continued till the 12th, the *fluor albus* being greatly diminished, and then gave up in consequence of the approaching fast. On the 25th December, I went to pay her a complimentary visit, when she complained of general indisposition, oppression of the stomach and breast, and with marked regret declined to defer drinking any wine or sweet spirits till the evening. Next morning, oppressed with headache after being intoxicated, she remained in bed till midday. She eat very little, but was very thirsty, resisting, however, the desire for drink, because I remained there the whole day. Next day, the 27th, I was invited by her husband, who concealed the true cause from me, to spend the day in his house; the lady was in low spirits. On the 28th, being invited, she reluctantly visited her husband's friends. On the 29th, because of the nausea which she felt, she requested an emetic; I gave her one grain of tartar emetic in four ounces of water, of which, on the following day, she took one spoonful, whereby she had two stools with *tenesmus.* On the 31st, she affirmed that she was much better, yet she eat nothing, was morose and sleepless. On 1st January, after dinner she was drunk. On the 2nd was merry, said she was well, and in the evening became quite drunk. On the 3rd, in consequence of intoxication, she withdrew from society. The same on the 4th. On the 5th, the husband with tears confessed that his wife was lying in her usual paroxysm of continued intoxication. Truly astonished at this unexpected event, I requested a full narrative, and received the previous details of his wife's past life. Having admitted that he entertained a hope when the paroxysm ceased during my treatment of her in the former month, he besought me to cure her of this habit by proper remedies.

I immediately went to the unfortunate woman. What did I see? A woman raving mad, with dishevelled hair, staring eyes, red face—the muscles of which, by their multifarious and rapid motion, presented, as it were, the image of the disturbed mind—joy, anger, rage, fury, succeeding each other by turns, gestures and expressions of which Messalina would have been ashamed. She who formerly appeared a respectable, amiable, and virtuous woman, now dressed in a tattered robe, sometimes addressed her husband in obscene language, sometimes immodestly assailed her domestics; at others, foully abused her son or those who attempted to prevent her from drinking. She received me with bursts of laughter, then told me various anecdotes of her husband, requested that I would drink with her, and entreated that I would order spirits to be produced. I listened in amazement; for three days she had eat almost nothing, but had drunk three goblets (each containing four liquid pounds) of spirits, and an unknown quantity of *siceræ dulcis et amaræ* (sweet and bitter drams).

Wherefore, that I might the better understand her manner of potation and its consequences, I desired spirits only to be given to her. Her pulse was sub-febrile and frequent; *coma vigil.* Every instant she asked for a little drink, which when obtained she would sing, laugh, and rejoice; but if it were refused, became enraged, tearing whatever she could lay her hands upon, and lashing herself into a fury. On the following days, 6th, 7th, 8th, 9th, and 10th of January, she remained in the same state, daily swallowing half a cupful. On the 11th, when her husband had refused her the desired quantity of liquor, she came to my house, and calling me her father and saviour, besought me for drink; but when she saw that I would not satisfy her, she returned home raging furiously. The husband, overwhelmed with distress, besought me to order an emetic to be mixed with the spirits. I therefore directed two grains of tartar emetic to be dissolved in six ounces of spirits, and two doses to be given at the interval of an hour. By these means, after two hours, with previous sickness and prostration of strength, she twice with difficulty vomited a small portion of limpid and viscous matter. She passed a restless night, had a rapid and spasmodic pulse, repeatedly vomited a little limpid urine, and was tormented with great thirst and heartburn. I prescribed for her spirits and water. On the 12th of January, the paroxysm continued, but the symptoms were not so serious. On the 13th, she was raving, poured invectives upon her husband, and committed other improprieties. She drank a goblet of spirits diluted with an equal quantity of water. On the 14th, by my permission, she was satiated with liquor, was happy and tranquil, singing the whole night. The necessity for making water was less frequent. She continued in nearly the same state daily until the 19th, having taken the same quantity of liquor. On the 20th, perceiving that the spirits were diluted with water, she threw down the cup. Fury, both uterine and vinous, being at the height, it was necessary for allaying the transports, to give her pure spirits, of which she drank two cups. In the evening, she began to talk thick (or stammer), then to be silent, her head hanging on her breast or shoulders; with frequent and deep sighs she deplored her wretched condition; after weeping, fell asleep, then suddenly awaking, felt sick, with no desire for drink, the pulse weak and almost vermicular; the prostration of strength was at its height.

On the 21st of January, being summoned at five, a.m., I saw her for hours vomit sour, corrosive, viscous matter. Whenever she became a little more in her senses, she shed tears and tore her hair, begging forgiveness from every one near her. The vomiting was followed by diarrhœa wth tenesmus, the alvine fæces being similar to those ejected by the mouth; dysentery, with pale urine; severe heartburn; burning thirst; coma; spasmodic twitches; succeeded by sleep for an hour, accompanied by profuse perspiration redolent of spirits. In this state of matters, I ordered for sustenance a decoction of the root of salep, and for drink, toast and water, with infusion of saffron and quassia, to be taken by spoonfuls. The night passed with frequent slumbers, distracted by frightful dreams. On the 22nd, she complained of violent headache; the pulse was feeble and rapid, yet not very irregular, the thirst not so intense, the hands shook, and the strength was prostrated. That these should now be restored by aromatic and stimulating remedies, the merest tyro in medicine would have concurred with me in thinking; but when from the very large class of such remedies I endeavoured to select the most appropriate, I remembered the recommendation by the immortal Linnæus of *serpyllum*, which has been recorded both verbally and in writing by Murray, the distinguished pupil of that illustrious man, as an antidote to headache arising from excess; I therefore prescribed a draught of serpyllum, saffron, and cardamoms. She passed a quiet night. On the following day, the 23rd, she was very much better, and appetite for food gradually, with sleep, returned. On the 24th, she blushed when she saw me: her mind was now evidently restored, and all the symptoms much mitigated. On the 25th, being much better, she had a too copious catamenia, with slight colic. From the 26th to the 29th, the menstruation was more moderate, appetite and sleep were good, the bowels were moved twice a day, the desire of micturition less frequent, without difficulty, and the water of a citron colour. On the 30th, she recounted to me her sayings and doings during the paroxysm, entreating me not to abandon her; wherefore I ordered the medicines above mentioned to be continued till the 10th of February, that the period of the anamethysis, which commenced about the beginning of the last month, might pass away during the continued use of the remedy; for since, after enormous abuse of spirits, and continuous raging intoxication for eighteen days, she recovered by the use of my prescription very rapidly, I thought it might be tried whether, by the continuous use of the same nerving and stomachic medicine, the oinomania itself might be put to flight.

Ten days having elapsed, on the 21st of February, when the period of the catamenia again returned, the patient herself, afraid of a new access, besought from me a remedy, and used the preceding during seven days. The menses appeared on the 25th, continuing moderately for four days. The *fluor albus* diminished.

Finally, as no symptom appeared at the next term of menstruation, or the usual paroxysm of oinomania preceded it, she discontinued the medicine. The menses flowed moderately at the accustomed time.

On the 18th of April, the husband being a little elevated, insisted that she should drink a small cup of strong liquor. At first she refused, but at length, yielding to his importunity, she drank it with reluctance. Shortly after, she became sick and vomited. A restless night ensued, and next day headache, heartburn, rumbling of the belly, and frequent desire to make water. Whereupon, dreading a return of her former complaint, she asked for and took the medicine. On the 20th, she felt better; on the 21st, was quite well; on the 24th, the menses flowed more copiously for four days; on the 30th, she gave up the medicine.

Thenceforward, as she was thoroughly convinced of the utility of this remedy, and desired to provide against any relapse into this pernicious habit, she made use of the same medicine for five days at each time of menstruation in the following months of May, June, July, and August. Five years have now elapsed, in which she has never been seen intoxicated or tipsy. Menstruation is always regular in time and character. The loathing to spirits which she once had by degrees disappeared, but after a year had gone by, having no inclination for spirits, she began to take a very little wine, principally on fasting days, for the sake of promoting digestion. Withdrawing from the dangerous intimacies which were the primary cause of her error, and ashamed of her previous life, she discharges the duties of wife, mother, and friend, beloved by all who know her.

I freely admit, that in this successful care of a most pernicious vice, I can ascribe nothing to myself; for I only studied how to remove the bad effects produced by the great abuse of spirits by a remedy that should sustain and restore the powers of the body, exhausted not only by the subtraction of proper aliment, but injured to an incredible extent by the continued absorption of a violent stimulus; and as I was embarrassed at the time by the use of compound medicines, I had recourse to something simple and suitable to my indications. The *serpyllum,* which in that country is burned with the object of increasing milk in cattle, being ready to hand, I gladly remembered the recommendation of Linnæus; besides, the saffron which, being an excellent antispasmodic, I added as a sedative, was on sale in the taverns; and next, the cardamoms, as a capital carminative. The result far exceeded my expectation; every symptom was diminished, and menstruation, which drunkenness at the preceding period had suppressed, returned at the usual time as the sign of right order restored in the body. Why not, therefore, continue a remedy so wholesome? The patient herself, subsequently anticipating by her own instinct the perception of the physician, for fear of a returning paroxysm, at every new period sought for the remedy, and, undeceived by her hopes, patiently took it for six months during the time of menstruation, which formerly was also that of drunkenness.

In the following year, 1814, a man addicted to liquor afforded me an opportunity for trying the same remedy in a similar case.

### Case II.

A. T., a man of forty, of sanguine temperament, middle size, hardy texture, healthy body, who had attained to the rank of a Titular Counsellor, married in his 25th year a girl more beautiful than chaste. Having no fortune of his own, and only a moderate salary, he was indebted for a more comfortable livelihood to commerce in which his wife trafficked; whence it happened that from the commencement of his matrimonial career the wife ruled her husband, and suffered him in nothing to contradict her; he therefore bore his wife's licentiousness, if not with equanimity, in silence, and dispelled his chagrin by the aid of Bacchus, at first chiefly using beer, at other times various strong liquors. But after indulgence for ten years, in that which I have named ephemeral, had led him to regular drunkenness, being deprived of his office for negligence and prosecuted at law, he fell from fresh grief into continued intoxication, which, at first maintaining a trimestrial type, generally occupied the space of a week. In that time an emetic infused in his liquor by a physician produced no beneficial result; for the paroxysm becoming more frequent, in another year assumed the bi-monthly, and in the third the monthly type. At the beginning of a fourth year of this disgraceful habit, when he found himself cast by the judges, endeavouring to destroy life by drink, he was restored from the apoplectic state into which he had thrown himself by repeated bleeding and the application of blisters and ice to the head; yet so, that from that time, as each new period of drunkenness returned, his mind became alienated, yet by the very paroxysm of anamethysis was restored. His physician afterwards, besides vegetable diet, administered laxative and moderate remedies, and prescribed for his drink *trifolium fibrinum spiritu elixatum* and strong coffee. This treatment did him so little good that the hands began to shake both during the paroxysm and the intermission, the mind to be dulled, the face inflamed, and the feet swollen.

On the 11th of January, 1814, when his wife applied to me for advice, I desired that I should be sent for after the paroxysm of anamethysis had commenced.

Accordingly, on the 13th January, I first visited the drunkard, whom I found with a face swollen and livid, eyes fixed, trembling in every limb, stupid, wandering, in a copious cold perspiration, with irregular spasmodic pulse. At night, imagining that he was taken into custody, he sought to go out of doors, and indeed, towards midnight, when the attendants were aleep, ran to the court and the garden, where, having fallen into a pit, he required to be taken out by the servants. The intense cold did not allay his madness, for he was with difficulty brought back to the house. Next day, 14th of January, I gave him *croci infusum spirituosum cum liquore anodyno.* During the day he was somewhat more tranquil, but at night every symptom presented itself anew. On the 15th I administered the same with the addition of serpyllum. Lucid intervals followed during the day, with efforts to vomit, and copious pale urine. At night the delirium was moderate, the tremor of the limbs less violent, short sleep, perspiration not so abundant.

In the three following days every symptom became gradually diminished, reason returned, he was oblivious of the past, slept at night, had a desire for food, the urine was more plentiful and of a citron colour, diarrhœa slight. On the 20th, the hæmorrhoidal flux for the first time appeared, and the colour of the face was natural. His wife, remarking that for fifteen years he had never been so cheerful and apparently sane, entreated that the medicine should be continued. On the 23rd, the swelling of the feet disappeared, the appetite returned with sleep; he detested spirits, but did not dislike beer. Prohibiting therefore this, as well as various kinds of compounded drinks, I recommended to him a small glass of white wine before and after dinner, and *oxyzythum* for his ordinary drink. Having continued the medicine till the 3rd of February, he called upon me on the 7th of that month for the purpose of thanking me, when he affirmed that he was quite recovered. But he consented, on the return of the moon, to resume the medicine.

In the following month of March, his cause having been decided, he went with his wife to the metropolis; but on his way to his native city, amid the greetings of relations and friends, the hæmorrhoidal flux ceased from frequent inebriation, and a species of melancholy began to return. The wife again requested my advice, and I sent him some of the medicine, which had the desired effect.

After some months, I saw the same person, who had returned with his wife to the provincial town for the purpose of undertaking some new employment, well and hearty; and heard, that two days after resuming the medicine, the hæmorrhoidal flux again flowed, and his sleep and liveliness of mind returned. I persuaded him henceforward to revert to the use of the medicine; and from his wife's silence I infer that he has been well ever since.

### Case III.

John Klepetin, a merchant of Perm, aged 40, of choleric-sanguineous temperament, middle size, and firm texture, fell into a habit of drinking in his 20th year; which from his 27th began to be continuous, and therefore had lasted for thirteen years. The intermissions of this habit never extended beyond six days; the accesses were of various lengths, the longest fifteen, the shortest seven days; so that, as he wrote to me, for thirteen years a week never elapsed without his being drunk. Yet, notwithstanding this prodigious depravity, he contracted no other disease beyond some very painful blind piles.

On the 3rd of January, 1814, when his wife called me in, I found him on the ground sitting on a mattress, filthy on every side with most fœtid fæces ejected from both ends. On seeing me, he endeavoured, but in vain, to rise to salute me; he therefore crawled upon his hands, embracing my feet and compelling his wife and children to prostrate themselves, with tears and stammering voice, implored me that I would cure him from the insanity of drinking. This having promised, he returned to his couch, and oblivious of all that he had just said as to his wretched condition, called for liquor. This when his wife refused, he began to groan, employing all the eloquence of which he was capable to prevail upon her to relent, and to weep; but never, to my astonishment, though steadily denied the liquor, was he moved to rage. Of all other drunkards whom I have seen, this man alone in his paroxysm was distinguished by extreme docility.

At the period of his accesses he daily swallowed two cups and a half of strong liquor; but if at any time beyond spirits he drank plain beer or bitters, he forthwith vomited and made an end of the anamethysis. Then that shorter intermission ensued, which used to succeed the paroxysm, terminated by the use of simple liquor.

There was this also remarkable in him, that during these thirteen years he had the most perfect recollection of everything that was said or done during the time of his paroxysm or intermission. And his wife informed me, that in the time of his accesses he constantly, in matters relating to his business, gave clear replies and unhesitating orders.

On the 4th of June, he began to use the medicine of saffron, serpyllum, cardamum, and star anise. An hour after the first dose he vomited with great effort a matter so acrid that it burned the fauces and œsophagus. In sixteen hours he vomited twenty-four times, and was purged eight times. Yet, after these excessive evacuations, when I visited him in the evening, I found his strength not much prostrated, but a burning thirst, very great heartburn, violent headache, pulse spasmodic, yet not too feeble. He loathed spirits, and to allay his thirst asked for cranberries and pickled cucumbers, which I allowed him to have. At intervals he slept. In consequence of the sickness which the smell of spirits induced, he was removed from his filthy bed to another. On 5th June, the medicine was continued. He vomited twice, but was purged fifteen times, the thirst was less, and the pain in the head and heart diminished. He had a desire for *crambidium* with pickled cucumbers. He slept for hours, frequently prayed, wept, and blessed the medicine. June 6th, the medicine was continued; no vomiting, the alvine excretions less frequent than on the preceding day, little thirst, fair appetite, pulse gentle, regular, and sufficiently strong. Sleep good. June 7, medicine continued, six stools, mind cheerful, sleep and appetite good, strength entire. Incredible aversion to spirits. June 8, micturition more frequent, perspiration during the night, the rest as on the preceding day. June 9, four stools, copious micturition and perspiration. June 10 and 19, bowels moved thrice daily, the abundant perspiration and micturition continued. With the daily use of the medicine until 9th July, the secretions gradually were diminished; finally, on the 10th July, as he felt that the mania for drink was almost expelled, he requested permission to discontinue the medicine.

His private affairs having been considerably injured by the protracted drunkenness of so many years, he took upon himself the care or inspection of those shops where spirituous liquors are sold. Remembering the saying, that "he who loves danger will perish in it," and fearing greatly for the man's health, I exhorted him that he would, at least, make me aware whenever he felt a desire to drink. But four months afterwards he told me a very different tale, viz., that he was compelled to resign his appointment, although a lucrative one, *because of the intolerable smell of the spirits*, and, what was far more disagreeable, *the frequent necessity of tasting them.*

For the three next years I had no occasion to see him. His aversion to spirits still continued, as was proved in the fourth year, when he went to the fair of Makari, in company with a familiar friend—a regular toper: for this man, during an access of intoxication having invited Klepetin, endeavoured to force him to join in his potations, by locking the door of the room. But when Klepetin insisted that he could not swallow spirits, thirty-two flagons of champagne were brought in, whereof each drank a half. Klepetin, after absorbing such a quantity of wine, being carried home drunk, snored the whole night. Next morning, when in addition to heartburn and frequent alarms, his strength was much reduced and all his joints ached, he caused himself to be put into a warm bath and shampooed. After coming from the bath, slaking his thirst with *oxyzythum* and appeasing his hunger with dried fish, he went to sleep and awoke quite well. After this excess his dislike to spirits was not so great as before, but he continued to avoid them. A letter, which I received from him two months ago, returning me warm thanks, mentions that he is in excellent health, and that he abstains from spirituous liquors.

Of the remaining 47 cases I give tables, in which are recorded the names of the patients—for the most part by initials only—the sexes, ages, position in society, temperament, predisposing and occasional causes of anamethysis, the duration, type, length of access, attendant symptoms, species and manner of intoxication, the use, beginning and end, sensible effects and success of the remedies applied.

Of these 50 oinomaniacal cases observed by me, there were—

| | |
|---|---|
| Of the stronger sex | 39 |
| Of the weaker | 11 |

Of men, whose ages were—

| | |
|---|---|
| Between 20 and 30, there were | 7 |
| „ 30 and 40, „ | 19 |
| „ 40 and 50, „ | 11 |
| „ 50 and 60, „ | 2 |
| Of 60 | 1 |
| Of 80 | 1 |

Of women, whose ages were—

| | |
|---|---|
| Between 20 and 30, there were | 2 |
| „ 30 and 40, „ | 8 |
| „ 40 and 50, „ | 1 |

Whence it appears that the stronger more than the weaker sex, and of the former, those between 30 and 40, are most addicted to drinking.

The continued causes of drunkenness seem to proceed—

| | |
|---|---|
| 1. From effeminate education, luxury, associating with tipplers, drinking, gambling, &c. | 27 |
| 2. From grief by reverse of fortune, injuries, shame, &c. | 8 |
| 3. From unsatisfied lust | 1 |
| 4. From tænia | 9 |
| Unknown causes | 5 |

Of the cases mentioned in these tables, I cured 48; from which are to be deducted 6, who, preferring to be intoxicated rather than to be cured, withdrew themselves from my care, and employed remedies either very sparingly or irregularly. Of the 42, those who were thoroughly cured and never relapsed, are 28; those temporarily cured, but who relapsed, are 14. By this the efficacy of the remedies which I administered is more than established. Who does not perceive that a disease, which generally proceeds from neglected and effeminate education, from mental weakness and depraved habits, may frequently be cured by the mere art of the apothecary? In order that all may be cured, besides the necessity that the depraved, depressed, and weak mind should be aided by corrective and strengthening medicines, the occasional causes must be removed, or, if that cannot be, impeded, lest the unhappy individual rush from one evil into a greater.

It is needless for me to comment longer, before gentlemen skilled in all medical knowledge, on the power and mode of action of the remedies which I administered. The most notable belong to the nervine or exciting, the anodyne and strengthening class. I employed the anti-hypochondriac and anti-hysterical method, because I considered that the rage and necessity for drinking lay in the hypochondria. This method seldom failed, and I therefore rely upon it the more, because the use of those nervine remedies was generally followed by prodigious but highly salutary evacuations, whereby the body, fed by an immoderate absorption of spirits, and the blood changed into *vappa*, is purified; and which finally, by the healthy operation of nature, set free, spontaneously abating, frequently leave a large appetite for nutritious food.

I have certainly found the unlearned use a variety of things whereby the rage for drinking may not only be extinguished, but even an invincible aversion to all sorts of spirituous liquors produced. Of these, some are nauseous and horrible, which even to name is abhorrent. But by those which I have described, I think, by God's blessing, a more easy and agreeable cure of a serious and almost irremediable evil may be effected.

### ADDITIONAL REMARKS.

(*From Letter of the Author, dated* 10*th December*, 1819.)

After I learned that my observations on Continuous Drunkenness had been sent to press, I thought that it might be useful to communicate to you some remarks which the distinguished Professor of Medicine at Rome, Dr. Bucciolotti, wrote to me on the 18th of April last, in order that, if agreeable, they may be added to my statement: for as it is very possible that others may have viewed the subject in the same manner as myself, therefore what I shall adduce for my own defence against the learned professor, may likewise have been said by others. As the letter of this distinguished physician is written in Italian, I shall translate his principal observations.

"Although the author (A. M. Salvatori) may have imagined himself to have been the first to describe oinomania as a distinct kind of disease, yet it cannot be denied that the ancient pathologists had not overlooked it, and had endeavoured to prevent, and to cure it as such. The most ancient nations studied to repress this pernicious habit by severe laws. Saturn forbade the use of wine to the Sabine women; Faunus, King of the Latins, continued the same law, and ordered his own wife, who had violated it, to be flogged with myrtle rods. Romulus prescribed the same law to the Roman matrons. In later times, the Romans punished drunken women with death. All wine was forbidden to the youths of Greece, until they had completed their eighteenth year, and from that age until they were forty it was diluted with water; in more advanced life they were permitted to drink a larger quantity and stronger, yet not to intoxication. The laws of Carthage prohibited the use of wine to their princes during their annual reign. The Persians used it in moderation for exciting genius and valour. Therefore even the most ancient people were above all things solicitous to meet this fatal vice by the most efficacious of all remedies—the interdiction of wine.

"Etmüller, the very learned medical author, who perhaps supplied Gaubius with the phalanx of evils arising from inebriety, says, 'The drunkard is attacked by vertigo, his eyes are strained, his ears ring with a wavy sound, his tongue stammers, his speech is incoherent, his breath is foul, his stomach is upset, his heart palpitates; there is a foolish and unmeaning gesticulation of his hands, his gait is unsteady and slipping; his nights are restless, with heavy snoring and raving dreams, accompanied with most filthy discharges from the belly and bladder, wherein he frequently wallows like swine in their dirt.' Of drunkenness, which the Latins call *crapula*, he gives this definition: 'A morbid product, injuring the action of the animal spirits, arising from the sulphur of wine too copiously imbibed.' Moreover, he enumerates accurately and concisely the symptoms and various effects of drunkenness, and distinguishes the qualities of the wines which, according to their inherent nature, more or less sulphureous, injure the vital spirits with different force. He then propounds a cure.

"Tachenius and Bechius go over the same ground, suggesting as a remedy herbs 'containing alkali, such as healing and all pot herbs,' for they separate the intoxicating power of the wine from its acid, which by these plants is absorbed and modified. They likewise recommend oily plants, as such qualify the acid by their fatness, and thus prevent intoxication.

"But Platerus attributes rather to acids the like power of correcting the narcotic influence of wine and preventing drunkenness; and he recommends *posca*—that is, pure wine mixed with vinegar—as an antidote. Langius extols emetics.

"Therefore, in seeking a cure for this disease, the ancients neglected nothing. But, if the merit of novelty may not be allowed to the author, no one will readily deny that he well deserves praise for a more perfect description of the disease, and a more learned and effective method of curing it, as well as one better adapted to the nature of the complaint."

To the remarks of this illustrious physician, I may be permitted to reply briefly. He seems to confound *drunkenness* with *habitual drunkenness*, the *abuse* of wine with the necessity for the *abuse*. In truth, what medical authors have written upon the subject I mentioned in the beginning of my essay in the works of Gaubius, which appeared to me concisely and eloquently to convey the opinions of previous writers, and to which Etmüller, Tachenius, Platerus, and others cited, add nothing: for indeed nothing else is added to general and special pathology from the observations of these authors, than the knowledge of the pernicious consequences of an abuse of strong liquors. Among these, indeed, Gaubius enumerates the necessity for drinking; but that, neither he nor the above-named distinguished writers, viewed as a peculiar species of disease. Most of them regarded anamethysis as a degrading habit, therefore rather a disease of the mind than a bodily ailment. But I have not only studied from much observation to depict as a most loathsome vice this insatiable desire for spirits and continuous imbibition, and the symptoms which attend and follow it, but also to arrive at the knowledge whence this desire and fatal necessity for drink may proceed, and how it may be destroyed; that is, how the desire, which from its effects is called oinomania and anamethysis, may be cured. And in this I do not see that I have been anticipated by any one. For it is one thing to cure a drunk or wine-gorged individual—that is, to destroy or moderate the effects of strong liquor; and besides these remedies, which are prescribed by Tachenius, Bechius, Platerus, and Langius, many other more effective are known to the public, such as cold applications to the head and the scrotum, draughts of cream, infusion of coffee, &c.;—but it is another to eradicate in drunkards that dire necessity for drink arising from bodily defects, which truly can be done no otherwise than by the cure of that ailment which is the cause of this insane furor, united with such a care of the feeble mind that may add to it necessary strength, or, if that cannot be done, may sustain and prevent it by some other mode from relapsing into the evil habit, and the same body, perhaps by due remedies in a great measure restored to health, again falling into its primitive debility. To this difficult work I have candidly opened the path which experience pointed out to me, but I am still ignorant that I have been forestalled by any older or more recent author.

Other abuses, which from daily habit become a necessity, appear to require similar treatment; one, namely, of mind and body together; for from daily habit the body is generally so altered, that the strongest intellect can scarcely keep it under control, and restrain it from its own debasement.

[The tables accompanying our author's papers are too extensive to be reproduced within moderate compass. We append, however, a synoptical specimen of the cases he records.]

SYNOPSIS OF FIFTY CASES OF CONTINUED, REMITTENT, AND INTERMITTENT INTOXICATION, EITHER CURED OR NOT CURED, BY A. M. SALVATORI.

1. *Number and Name;* 2. *Sex, Age, Civil Rank, Temperament;* 3. *Causes; (a) Predisposing, (b) Occasional;* 4. *Duration;* 5. *Type;* 6. *Length of the Accesses;* 7. *Attendant Symptoms;* 8. *Liquor: its kind and daily quantity in the Access;* 9. *Species;* 10. *When begun to be used;* 11. *Continuation of Use;* 12. *Sensible Effects;* 13. *Success.*

Case I.—1, M. N.: 2, woman, age 31, noble, sanguine temperament: 3 (*a*) idleness, (*b*) intimacy with drunkards: 4, ten years. 5, Menstrual, during the period of the catamenia: 6, various, as much as sixteen days: 7, Nymphomania, excessive menstruation, repentance, shame after the access: 8, Spirits (frūmenti sicera): 1 goblet (cantharus i. j$\beta$. ii.): 9, Serpyllum, saffron, cardamoms: 10, 1813, January 22, two days after the end of the paroxysm: 11, thirty-five days repeated monthly till August: 12, weakening of the succeeding symptoms, menstruation: 13, health thoroughly restored.

Case II.—1, A. J. J., 2, man, age 40, in a public office, sanguine temperament: 3, (*a*) domestic affliction, ten years' drunkenness, (*b*) public disgrace: 4, three years: 5, trimestrial at first, then bimestrial, at last monthly: 6, seven days: 7, dulness of mind and body: 8, spirits: 9, serpyllum, saffron, anodyne liquor: 10, 1814, January 14, at beginning of the paroxysm: 11, twenty-one days, afterwards repeated twice: 12, weakness, diarrhœa, scanty urine, hæmorrhoidal flux: 13, health restored.

Case III.—1, J. N.: 2, man, age 45, serf, choleric-melancholic-sanguineous temperament: 3, 4, fifteen years: 5, irregular: 6, various: 7, stupidity during intermission, fury during access: 8, at first beer then spirits: 9, serpyllum, saffron, star-anise: 10, 1814, March 5: 11, twenty-one days: 12, nausea, vomiting, diarrhœa, thirst, aversion to spirits, micturition, perspirations: 13, health restored, great aversion to spirits.

Case IV.—1, John Klepetin: 2, man, age 40, merchant, choleric-sanguineous temperament: 3, (*a*) unsteadiness, (*b*) ephemeral intoxication: 4, thirteen years: 5, irregular, longest intermission 6 days: 6, from seven to fifteen days: 7, painful blind piles: gentle disposition, prone to repentance: 8, spirits to the extent of ii. $\beta$. goblets: 9, serpyllum, saffron, star-anise: 10, 1814, June 4: 11, thirty-six days: 12, vomiting of acrid matters twenty-four times, horror of spirits, thirst, diarrhœa beyond fifty stools, micturitions, perspirations: 13, complete health, extreme aversion to spirits.

Case V.—1, J. N.: 2, man, age 50, gardener, serf, choleric-sanguineous temperament: 3, (*b*) intimacy with bad companions: 4, twenty-five years; in vain attempted to be cured by many physicians: 5, irregular: 6, seven days: 7, refused medical asssistance as unavailing: 8, spirits to the extent of two goblets: 9, serpyllum, saffron: 10, 1814, June 13: 11, four days: afterwards refused to be compelled to take it: 12, none; he got drunk while using the medicine: 13, none.

Case VI.—1, J. J. Z.: 2, man, age 30, captain, choleric-sanguineous-melancholic temperament: 3, (*a*) sedentary life, adverse fortune, rage for gambling, (*b*) losses at play, censures of his superior officers: 4, three years: 5, monthly: 6, at first seven, afterwards ten days: 7, most copious vomiting succeeding each access, diarrhœa, excessive perspirations: 8, brandy (vini sicera): 9, serpyllum, saffron, star-anise, stomachic tincture and rhubarb in sherry: 10, 1814, April 6, the day before access: 11, fourteen days, stomachic tincture, and twenty-five days star-anise: repeated monthly both before and after recrudescence: 12, vomiting, stools, aversion to spirits, micturition, perspirations, weakening of preceding symptoms: 13, for the time complete. Imperfect by reason of rage for drink, losses at play, &c.

[*Serpyllum* in the combinations already mentioned, but chiefly with absinthium, and occasionally with valerian, was used in all the cases of which the results are recorded.]

